# Unveiling the synergistic power of 3-hydrazinoquinoxaline-2-thiol and vancomycin against MRSA: An *in vitro* and *in silico* evaluation

**DOI:** 10.17305/bb.2025.11886

**Published:** 2025-04-01

**Authors:** Ohood S Alharbi, Mohanned Talal Alharbi, Mazen A Ismail, Ahmad M Sait, Mohammed Mufrrih, Wafaa Alhazmi, Bandar Hasan Saleh, Manal A Zubair, Noha A Juma, Noof R Helmi, Hatoon A Niyazi, Hanouf A Niyazi, Hussam Daghistani, Taghreed Shamrani, Waiel S Halabi, Abdelbagi Alfadil, Hisham N Altayb, Karem Ibrahem

**Affiliations:** 1 Department of Microbiology and Parasitology, Faculty of Medicine, Umm Al-Qura University, Makkah, Saudi Arabia; 2 Department of Basic Medical Sciences, College of Medicine, University of Jeddah, Jeddah, Saudi Arabia; 3 Department of Medical Education, Faculty of Medicine, King Abdulaziz University, Jeddah, Saudi Arabia; 4 Department of Medical Laboratory Sciences, Faculty of Applied Medical Sciences, King Abdulaziz University, Jeddah, Saudi Arabia; 5 Regenerative Medicine Unit, King Fahd Medical Research Center, King Abdulaziz University, Jeddah, Saudi Arabia; 6 Special Infectious Agents Unit BSL-3, King Fahd Medical Research Center, King Abdulaziz University, Jeddah, Saudi Arabia; 7 Department of Clinical Microbiology and Immunology, Faculty of Medicine, King Abdulaziz University, Jeddah, Saudi Arabia; 8 Department of Clinical Microbiology Laboratory, King Abdulaziz University Hospital, Jeddah, Saudi Arabia; 9 Department of Clinical Biochemistry, Faculty of Medicine, King Abdulaziz University, Jeddah, Saudi Arabia; 10 Food, Nutrition and Lifestyle Unit, King Fahd Medical Research Centre, King Abdulaziz University, Jeddah, Saudi Arabia; 11 Department of Optometry, Faculty of Applied Medical Sciences, University of Jeddah, Saudi Arabia; 12 Centre of Research Excellence for Drug Research and Pharmaceutical Industries, King Abdulaziz University, Jeddah, Saudi Arabia; 13 Department of Biochemistry, Faculty of Science, King Abdulaziz University, Jeddah, Saudi Arabia

**Keywords:** MRSA, vancomycin, 3-Hydrazinoquinoxaline-2-thiol, 3HL, combination therapy, *in silico* analysis

## Abstract

Methicillin-resistant *Staphylococcus aureus* (MRSA) is a major pathogen causing infections ranging from skin disorders to severe conditions like infective endocarditis. Its evolving resistance, including resistance to β-lactams and last-resort antibiotics, such as vancomycin, daptomycin, and linezolid, necessitates alternative therapies. This study investigates the synergistic efficacy of vancomycin and 3-hydrazinoquinoxaline-2-thiol (3HL) against 23 clinical MRSA isolates. Susceptibility testing was performed using broth microdilution and checkerboard assays, while *in silico* analyses assessed interactions between vancomycin and 3HL. Vancomycin exhibited minimum inhibitory concentrations (MICs) ranging from 0.25 to 1 µg/mL, whereas 3HL showed higher MICs of 16–32 µg/mL. Synergistic interactions were confirmed via checkerboard assays, with fractional inhibitory concentration index (FICI) values between 0.236 and 0.5, indicating enhanced vancomycin efficacy. Notably, vancomycin MICs decreased significantly when combined with 3HL. *In silico* docking revealed interactions with penicillin-binding protein 2a (PBP2a), suggesting promising therapeutic potential. Vancomycin exhibited superior docking scores (−8.9 kcal/mol) and stabilizing hydrogen bonds, effectively targeting key protein grooves. Both compounds demonstrated potential for overcoming PBP2a’s structural occlusions, suggesting their role in combating β-lactam-resistant strains through targeted protein inhibition and structural stabilization.

## Introduction

*Staphylococcus aureus* (*S. aureus*) is a versatile pathogen responsible for infections ranging from superficial skin conditions to severe diseases such as infective endocarditis [1]. Its adaptability has made it a significant contributor to antimicrobial resistance (AMR) [2]. Initially resistant to penicillin due to β-lactamase production, *S. aureus* later developed resistance to most β-lactams by acquiring the mecA gene, which encodes penicillin-binding protein 2a (PBP2a) [3, 4]. Alarmingly, resistance now extends to last-resort antibiotics, including vancomycin, daptomycin, and linezolid, complicating treatment [5]. This growing resistance underscores the need for alternative strategies, such as combination therapies and novel inhibitors, to combat *S. aureus*-associated infections effectively [6]. Vancomycin, the gold standard for treating infections caused by Gram-positive bacteria, including methicillin-resistant *S. aureus* (MRSA), has notable limitations despite its efficacy [7]. Its bactericidal activity is relatively low compared to other antibiotics, leading to slower bacterial elimination and prolonged therapy durations, which increase the risk of complications [8]. A key challenge is its inability to effectively target biofilms when used alone [9]. Biofilms—structured bacterial communities encased in a protective extracellular matrix—are notoriously difficult to eradicate due to their reduced susceptibility to antimicrobials [10]. Additionally, vancomycin resistance is increasingly reported in clinical settings, threatening its continued effectiveness as a monotherapy [8]. These challenges highlight the urgent need for combination therapies or alternative approaches, particularly for infections involving biofilms or resistant strains.

Combination therapy offers several significant advantages over monotherapy, even when a single drug demonstrates effective activity [11]. By employing two synergistic drugs, the required dose of each can often be reduced, minimizing dose-dependent toxicity and improving the overall safety profile [12]. This is particularly beneficial for infections requiring prolonged treatment or for vulnerable patient populations where high drug toxicity is a major concern [13]. Furthermore, combination therapy reduces the risk of resistance development, as pathogens face multiple simultaneous mechanisms of attack, making adaptation and survival more difficult [14]. Additionally, synergistic drug combinations enhance bactericidal activity, leading to faster and more effective pathogen elimination [15]. This is especially crucial in severe or complicated infections, where rapid bacterial clearance can significantly impact patient outcomes [16]. Moreover, combination therapy improves drug delivery and penetration into challenging infection sites, such as biofilms or poorly vascularized tissues, where monotherapy may fail to achieve adequate concentrations [17]. Collectively, these benefits make combination therapy a powerful strategy in combating infections, particularly in an era of rising AMR and increasingly complex infectious diseases. *In silico* methods and molecular docking play a crucial role in modern drug discovery and development [18]. These computational techniques offer several key advantages. *In silico* approaches can predict and analyze biological targets by mining databases, identifying conserved regions, and evaluating their potential as druggable sites [19, 20]. Docking studies simulate interactions between potential drug molecules and their targets, revealing binding affinities, interaction sites, and key residues involved, which aids in understanding the mechanism of action at the molecular level [21, 22]. Traditional *in vitro* and *in vivo* screening of drug candidates can be expensive and time-consuming [23]. In contrast, *in silico* methods enable the rapid screening of thousands of compounds, significantly reducing resource requirements [24]. Docking results also guide the rational design of new compounds by optimizing binding affinities and enhancing target selectivity, leading to more effective drugs with fewer side effects [25]. Additionally, docking studies help identify potential off-target effects, aiding in the development of more specific molecules—a crucial advantage for personalized medicine approaches [26]. Recently, 3-hydrazinoquinoxaline-2-thiol (3HL) has emerged as a promising compound with notable antimicrobial properties. Its efficacy has been demonstrated not only against bacterial pathogens but also against fungal species such as *Candida*, highlighting its broad-spectrum potential [27, 28]. In addition to its inherent antimicrobial activity, studies have shown that 3HL can synergize with penicillin to enhance its effectiveness against MRSA, underscoring its potential role in combination therapy [28]. However, despite these demonstrated benefits, no research to date has explored the efficacy of 3HL in combination with vancomycin, the standard therapy for MRSA infections. This represents a significant gap in our understanding of how 3HL might complement vancomycin’s bactericidal mechanisms to overcome resistance challenges. Therefore, this study aims to: (1) evaluate the synergistic potential of 3HL and vancomycin against MRSA through *in vitro* antimicrobial assays, (2) investigate the mechanism of interaction using *in silico* molecular docking analysis, and (3) assess the impact of this combination on key MRSA resistance targets. These findings could pave the way for innovative therapeutic strategies against this formidable pathogen.

## Materials and methods

### Bacterial strains and growth conditions

Clinical isolates of MRSA were obtained from the Microbiology Department of King Abdulaziz University Hospital (KAUH). All isolates were confirmed using standard microbiological methods and stored at −80 ^∘^C in 15% glycerol until further use. For all experiments, the isolates were cultured on blood agar or Mueller–Hinton agar and incubated at 37 ^∘^C under aerobic conditions.

### Determination of minimum inhibitory concentration (MIC)

MICs of vancomycin and 3HL were determined using the broth microdilution method, following Clinical and Laboratory Standards Institute (CLSI) guidelines [29, 30]. To prepare the initial concentrations of vancomycin and the quinoxaline derivative, as well as their serial dilutions, the equation C_1_V_1_ ═ C_2_V_2_ was applied. This method ensures precise preparation of the desired concentrations for MIC and FIC studies. A volume from the stock solution was diluted with Mueller–Hinton Broth (MHB), and serial two-fold dilutions were performed to obtain a range of concentrations. Then, 100 µL from each well was transferred to the next, creating a concentration gradient across the plate. Each compound was serially diluted in MHB and added to 96-well plates containing standardized bacterial suspensions at a final concentration of ∼5 × 10^5^ CFU/mL. Plates were incubated at 37 ^∘^C for 24 h, and the MIC was recorded as the lowest drug concentration that inhibited visible growth [5]. To ensure accuracy and reliability, appropriate controls were included in all experiments. A negative control (media alone) confirmed sterility, while a positive control (bacteria in media without antibiotics) verified bacterial viability. These controls ensured that growth inhibition was solely due to the antimicrobial activity of the tested compounds. For synergy assessment, the MIC of each drug—vancomycin and 3HL—was determined individually and in combination using the checkerboard assay. Monotherapy for each drug was tested separately, and the results were compared to combination therapy.

### Checkerboard assay for combination studies

The interaction between vancomycin and the 3HL was evaluated using a checkerboard microdilution assay. Serial dilutions of vancomycin were prepared along the horizontal axis of a 96-well plate, while dilutions of the 3HL were prepared along the vertical axis. Each well contained a combination of both compounds in varying concentrations, along with a bacterial inoculum of ∼5 × 10^5^ CFU/mL. Plates were incubated at 37 ^∘^C for 24 h.

Fractional inhibitory concentration index (FICI) was calculated using the formula:

FICI ═ (MIC of drug A in combination ÷ MIC of drug A alone) + (MIC of drug B in combination ÷ MIC of drug B alone) [14].

The interaction was interpreted as:

Synergy: FICI ≤ 0.5

Additive: 0.5 < FICI ≤ 1

Indifference: 1 < FICI ≤ 4

Antagonism: FICI > 4.

### *In silico* analysis

In this study, *in silico* methods were used to assess the potential synergistic effects of vancomycin and 3HL against PBP2a from MRSA. The crystal structure of PBP2a in complex with piperacillin at the active site (PDB ID: 6H5O) was downloaded from the Protein Data Bank (PDB) (https://www.rcsb.org/structure/6H5O). The 3D structures of vancomycin (ID: 14969) and 3HL (ID: 781248) were obtained from the PubChem database.

Before docking, the crystal structures were prepared by adding hydrogen bonds, removing water molecules from the protein, and performing energy minimization using the Maestro tool (2021). The SiteMap tool in the Maestro interface was used to predict active sites in PBP2a. Extra Precision docking in Maestro was employed to analyze potential interactions between the compounds and the protein’s active site. Additionally, MM-GBSA analysis was conducted to estimate the binding free energy (ΔG) of the complexes. The resulting complexes were analyzed for bond types, bond lengths, and interactions between the compounds and the protein using PLIP (https://plip-tool.biotec.tu-dresden.de/plip-web/plip/index). The PyMOL molecular graphics system (v2.5.8) was used to visualize the 3D interactions.

### Interpretation of results

All experiments were performed in triplicate, the average was calculated and results were expressed as the mean MIC and FICI values.

## Results

### MICs of vancomycin and 3HL against MRSA strains

The MIC values for vancomycin against 23 MRSA strains ranged from 0.25–1 µg/mL, with most strains exhibiting MICs of 0.5 or 1 µg/mL. In contrast, the MIC values for 3HL were consistently higher, ranging from 16 to 32 µg/mL. Notably, vancomycin showed lower MIC values, indicating greater potency against MRSA compared to 3HL. Strain MRSA 7 had the lowest MIC for vancomycin (0.25 µg/mL), while MRSA 105 and MRSA 106 displayed MICs of 0.5 and 1 µg/mL, respectively. The uniformity of 3HL MICs (primarily 32 µg/mL) suggests limited variability in its activity. These findings highlight distinct susceptibility patterns between vancomycin and 3HL against MRSA (Table 1).

**Table 1:** Interaction between vancomycin and 3HL against MRSA

**No**	**MRSA number**	**MIC Van** **µg/mL**	**MIC 3HL** **µg/mL**
1	105	1	16
2	104	0.5	16
3	95	1	16
4	92	1	32
5	75	1	32
6	106	0. 5	16
7	101	1	32
8	98	0.5	32
9	97	1	32
10	100	0.5	32
11	109	1	32
12	7	0.25	32
13	80	1	16
14	92	1	32
15	73	1	32
16	54	1	32
17	34	0.5	32
18	1	0.5	32
19	2	0.5	32
20	3	0.5	32
21	4	0.5	32
22	11	0.5	32
23	9	0.5	32

The strains are listed by their identification numbers along with the corresponding MICs in µg/mL. The data provides an overview of the antimicrobial susceptibility of MRSA strains to the tested agents. MRSA: Methicillin-resistant *Staphylococcus aureus*; MIC: Minimum inhibitory concentration; 3HL: 3-hydrazinoquinoxaline-2-thiol.

The interaction between vancomycin and 3HL was evaluated against 23 clinical MRSA isolates using checkerboard assays to assess their combined effects. The FICI values for the tested isolates ranged from 0.236 to 0.5, with an average FICI of 0.332, indicating a strongly synergistic interaction. Notably, vancomycin’s MIC values decreased significantly in the presence of 3HL, enhancing its potency. For example, vancomycin MICs dropped from 1 µg/mL to as low as 0.06 µg/mL in several isolates. Most isolates exhibited FICI values below 0.5 (Table 2), confirming synergy, while a few had values near 0.5. No antagonism was observed. Although slight variations in FICI were noted across isolates, the overall trend supported a synergistic interaction, particularly in isolates 101, 92, and 54, which consistently displayed FICI values within the synergistic range. This study highlights the potential of 3HL derivatives to enhance vancomycin’s antimicrobial activity against MRSA and underscores the need for further investigation to optimize this combination for clinical application.

**Table 2:** FICI values and corresponding interaction interpretations for vancomycin and *3HL* against various MRSA strains

**No**	**MRSA strain number**	**FICI**	**Interaction**
1	105	0.437	Synergy
2	104	0.450	Synergy
3	95	0.360	Synergy
4	92	0.342	Synergy
5	75	0.373	Synergy
6	106	0.310	Synergy
7	101	0.350	Synergy
8	98	0.375	Synergy
9	97	0.350	Synergy
10	100	0.332	Synergy
11	109	0.332	Synergy
12	7	0.346	Synergy
13	80	0.360	Synergy
14	92	0.375	Synergy
15	73	0.236	Synergy
16	54	0.395	Synergy
17	34	0.290	Synergy
18	1	0.370	Synergy
19	2	0.413	Synergy
20	3	0.335	Synergy
21	4	0.352	Synergy
22	11	0.342	Synergy
23	9	0.332	Synergy

FICI values ≤0.5 indicate synergy, while values between 0.5 and 1.0 suggest an additive effect. This table highlights the predominant synergistic interactions between the two agents across the tested strains. MRSA: Methicillin-resistant *Staphylococcus aureus*; FICI: Fractional inhibitory concentration index; 3HL: 3-hydrazinoquinoxaline-2-thiol.

### Vancomycin and 3HL predicted to efficiently inhibit the active and allosteric sites of PBP2a

**Figure 1.**
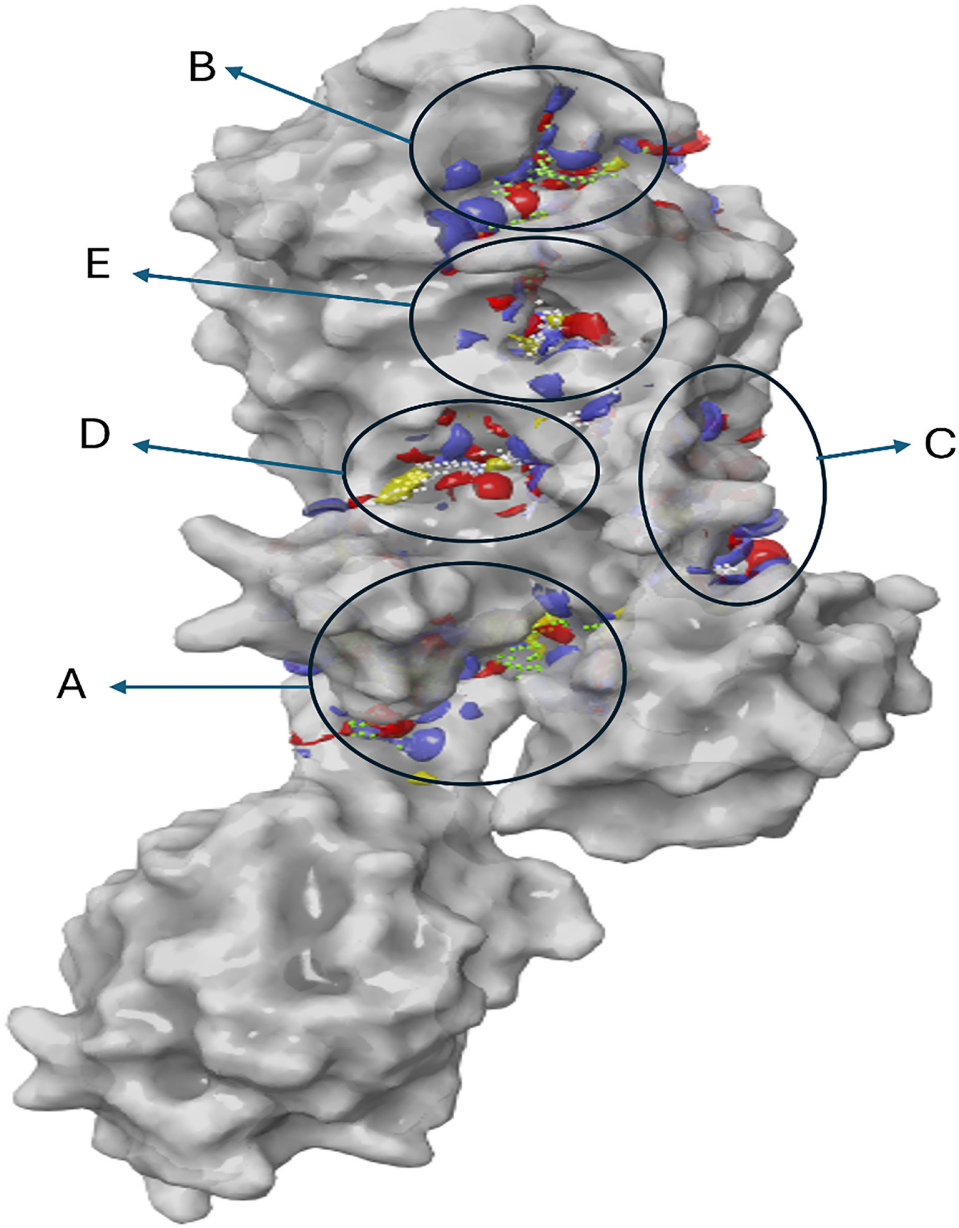
**Modeled 3D structure of PBP2a from MRSA showing the pockets, the active site is indicated by “A,” while the allosteric site is indicated by “B.”** While C, D, and E are other binding sites identified by SiteMap. The molecular interaction fields (yellow surface indicates hydrophobic, blue surface indicates hydrogen bond donor, red indicates hydrogen bond acceptor), and site-points (white spheres). MRSA: Methicillin-resistant *Staphylococcus aureus*; PBP2a: Penicillin-binding protein 2a.

**Figure 2.**
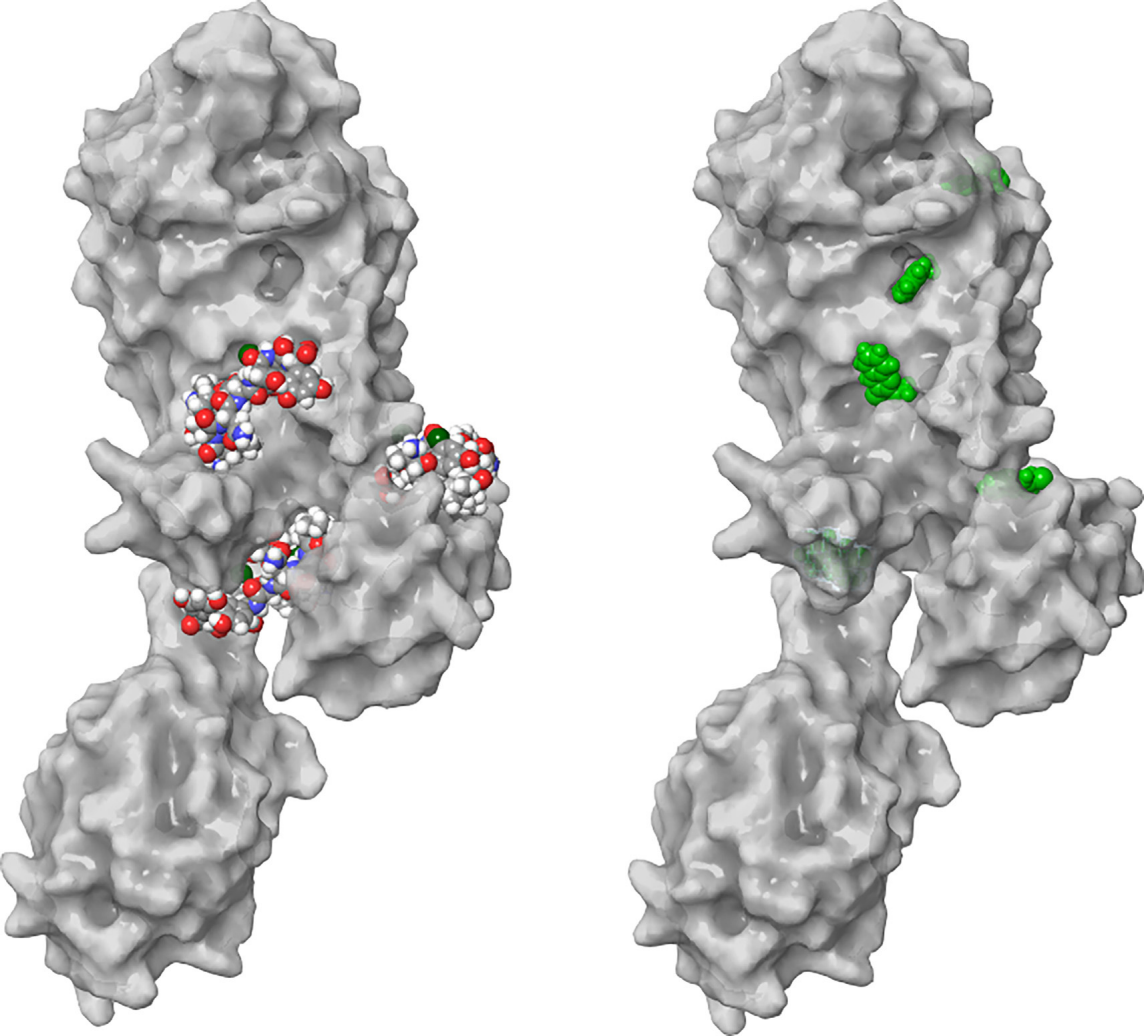
**Interaction of PBP2a from MRSA and (A) vancomycin and (B) 3HL.** Vancomycin interacted with sites A, C, and D as shown in Figure 1. The 3HL interacted with all five sites. The precise amino acids involved in each site are presented in Table 4 and Figure 3. PBP2a: Penicillin-binding protein 2a; 3HL: 3-hydrazinoquinoxaline-2-thiol; MRSA: Methicillin-resistant *Staphylococcus aureus*.

In this study, an *in silico* approach was used to predict potential inhibitors of PBP2a from MRSA, a protein essential for cell wall biosynthesis [31]. Screening of protein pockets identified five binding sites, most of which had Dscore values above 1.0 Å, except for site 5 (0.756) (Table 3, Figure 1). In general, pockets with Dscore values >0.98 considered druggable [32]. The known active site of PBP2a is indicated by “B” in Figure 1, while the allosteric site is marked as “A” [17, 33]. It has been proposed that the active site of PBP2a cannot be inhibited by β-lactams due to the presence of protective loops surrounding this region [17]. However, blocking the allosteric site has proven effective in treating resistant bacteria, as it triggers the opening of the active site, ultimately leading to its inhibition [17]. In this study, both vancomycin and 3HL effectively blocked the active and allosteric sites, as shown in Table 3 and Figure 2. Vancomycin (ID: 14969) exhibited the best docking score of −8.9 kcal/mol and a ΔG bind of −56 kcal/mol when interacting with the allosteric site (A). Additionally, it effectively blocked two other grooves (C and D) with docking scores of −7.8 and −10.8, respectively. These findings suggest that vancomycin may promote the opening of the protein’s active site and enhance its stability, preventing twisting and closure. Meanwhile, 3HL interacted with the active site, yielding a docking score of −4.9 kcal/mol and a ΔG bind of −37 kcal/mol (Table 3).

**Table 3:** Docking scores and MM GBSA dG bind of vancomycin (ID: 14969) and 3HL (ID: 781248) with different grooves in PBP2a

**Site**	**Dscore**	**Volume**	**ID**	**XP docking**	**MM GBSA dG bind**
A	1.018	527	14969	−8.9	−56
			781248	−3.9	−27
B	0.991	417	14969	−	−
			781248	−4.8	−37
C	1.011	375	14969	−7.8	−40
			781248	−3.3	−22
D	1.005	251	14969	−10.8	−50
			781248	−3.9	−31.9
D	0.756	151	14969	−	−
			781248	−3.1	−16.4

3HL: 3-hydrazinoquinoxaline-2-thiol; PBP2a: Penicillin-binding protein 2a.

**Figure 3.**
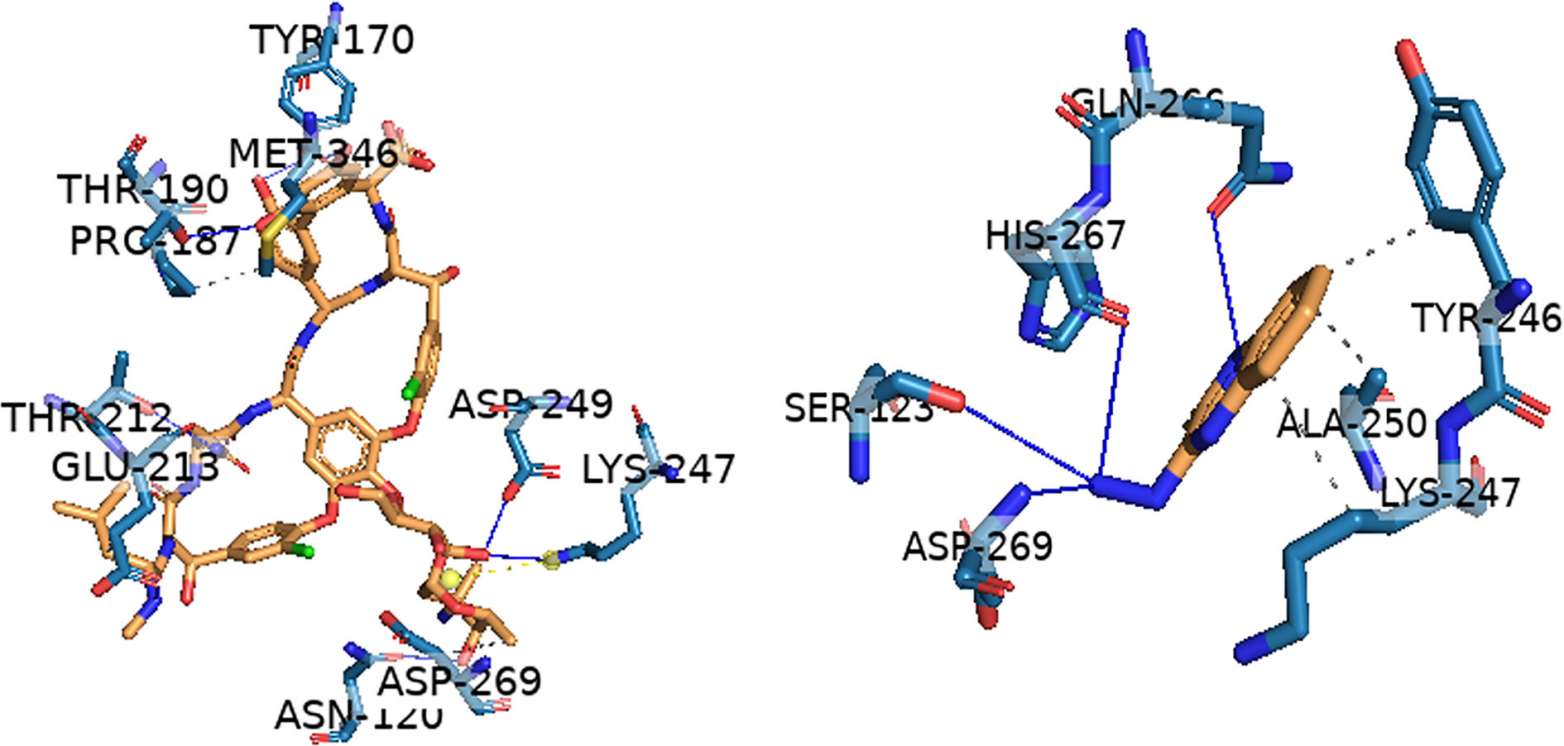
**3D interaction of vancomycin (ID: 14969) and *3*HL (ID: 781248) and with different grooves in PBP2a, the grooves are indicated by numbers as shown in Figure 1.** PBP2a: Penicillin-binding protein 2a; 3HL: 3-hydrazinoquinoxaline-2-thiol.

Table 4 and Figure 3 summarize the interacting residues of various compounds within different grooves of PBP2a. Vancomycin interacts with multiple residues, including ASN, TYR, THR, and GLU, across different grooves. It exhibits strong interactions, with donor-acceptor distances ranging from 1.67 to 3.45 Å. Meanwhile, 3HL (ID: 781248) interacts with residues, such as SER, GLN, HIS, LYS, and ASN in various grooves. Vancomycin specifically interacts with residues like ASN606 and THR373 (within or near the 594–603 region) [17], potentially aiding in overcoming structural distortion by forming stable hydrogen bonds (e.g., 1.87 Å with ASN606). This interaction may contribute to vancomycin’s ability to open the active site by stabilizing its structure and preventing loop-mediated occlusion.

**Table 4:** Interacting residues of vancomycin (ID: 14969) and 3HL (ID: 781248) and with different grooves in PBP2a

**Compound**	**Index**	**Residue**	**AA**	**Distance H-A**	**Distance D-A**	**Donor angle**	**Donor atom**	**Acceptor atom**
1_14969	1	120A	ASN	1.96	2.89	161.07	10311 [O3]	1981 [O2]
	2	170A	TYR	2.42	3.19	136.57	2777 [O3]	10326 [O2]
	3	190A	THR	2.43	3.02	119.29	3113 [O3]	10328 [O2]
	4	212A	THR	1.91	2.88	157.56	10340 [Nam]	3481 [O3]
	5	213A	GLU	1.76	2.78	177.59	10339 [N3]	3497 [O3]
	6	247A	LYS	2.28	3.03	129.45	4040 [N3+]	10312 [O3]
	7	249A	ASP	1.79	2.74	164.88	10312 [O3]	4073 [O-]
	8	346A	MET	1.78	2.72	161.79	10331 [O2]	5605 [O2]
1_781248	1	123A	SER	3.47	3.86	107.12	2035 [O3]	10310 [N3]
	2	266A	GLN	2.83	3.33	111.13	10307 [Nam]	4359 [O2]
	3	267A	HIS	3	3.93	151.65	10310 [N3]	4372 [O2]
	4	269A	ASP	2.71	3.71	171.49	4401 [Nam]	10309 [Npl]
2_781248	1	420A	TYR	2.17	3.18	173.27	10310 [N3]	6797 [O3]
	2	557A	HIS	3.08	3.91	134.71	8955 [Nar]	10308 [N2]
	3	616A	ALA	3.26	4.05	135.42	9880 [Nam]	10308 [N2]
	4	617A	SER	3.02	3.76	135.56	9895 [O3]	10310 [N3]
3_14969	1	163A	GLU	2.14	3.09	167.89	10314 [O3]	2667 [O2]
	2	165A	SER	2.34	2.82	110.22	2703 [O3]	10312 [O3]
	3	189A	LYS	2.22	3.14	150.99	3094 [N3+]	10314 [O3]
	4	193A	LYS	1.81	2.81	171.81	3168 [N3+]	10321 [O3]
	5	195A	ASP	3.25	4.08	140.99	10338 [Nam]	3206 [O.co2]
	6	196A	GLU	1.95	2.65	129.52	3218 [O3]	10328 [O2]
	7	196A	GLU	1.67	2.65	176.5	10328 [O2]	3218 [O3]
	8	197A	TYR	2.13	3.12	161.85	10339 [N3]	3237 [O3]
	9	350A	SER	3.06	3.55	110.62	5664 [Nam]	10312 [O3]
	10	350A	SER	2.77	3.52	135.82	5669 [O3]	10309 [O3]
	11	352A	GLU	1.88	2.87	160.97	10332 [N3]	5696 [O.co2]
	12	353A	GLU	2.23	3.15	159.83	10312 [O3]	5711 [O.co2]
3_781248	1	192A	LYS	3.08	3.95	145.74	3138 [Nam]	10308 [N2]
	2	193A	LYS	2.17	3.14	160.61	10309 [Npl]	3163 [O2]
	3	193A	LYS	2.47	3.12	121.92	3168 [N3+]	10310 [N3]
	4	195A	ASP	1.91	2.76	138.61	10310 [N3]	3206 [O.co2]
4_14969	1	225A	HIS	2.07	3	161.49	10314 [O3]	3675 [O2]
	2	237A	GLU	1.84	2.78	153.25	10339 [N3]	3859 [O.co2]
	3	240A	GLN	2.38	3.36	164.29	3915 [Nam]	10325 [O2]
	4	256A	GLY	3.45	4.04	122.21	10311 [O3]	4177 [O2]
	5	256A	GLY	2.43	3.17	129.13	4174 [Nam]	10308 [O3]
	6	340A	TYR	1.96	2.82	146.82	5509 [O3]	10315 [O3]
	7	358A	THR	2.07	2.95	150.86	10331 [O2]	5800 [O3]
	8	365A	LEU	2.15	2.86	129.54	10315 [O3]	5912 [O2]
	9	367A	ASN	2.37	3.26	147.12	5954 [Nam]	10312 [O3]
	10	370A	GLN	2.78	3.2	107.42	10312 [O3]	6010 [O2]
	11	370A	GLN	3.24	4.05	138.44	6011 [Nam]	10309 [O3]
4_781248	1	340A	TYR	2.16	2.88	130.94	11018 [O3]	20614 [N3]
	2	365A	LEU	1.91	2.81	144.73	20614 [N3]	11823 [O2]
	3	367A	ASN	3.11	3.99	146.24	11904 [N3]	20611 [Nam]
	4	367A	ASN	3.64	3.99	102.3	11908 [N3]	20611 [Nam]
	5	367A	ASN	3.46	4.09	122.37	11893 [N3]	20612 [N2]
	6	370A	GLN	1.86	2.71	139.34	20611 [Nam]	12016 [O2]
5_14969	1	373A	THR	2.92	3.85	162.41	6058 [O3]	10309 [Npl]
	2	606A	ASN	1.87	2.81	150.44	10309 [Npl]	9731 [O2]
	3	606A	ASN	2.75	3.51	131.98	9732 [Nam]	10310 [N3]

Bottom of Form. 3HL: 3-hydrazinoquinoxaline-2-thiol; PBP2a: Penicillin-binding protein 2a.

## Discussion

This study revealed that vancomycin exhibited potent activity against *S. aureus* clinical MRSA isolates, with MIC values ranging from 0.25 to 1 µg/mL. However, 3HL displayed relatively higher MICs (16–32 µg/mL). Checkerboard assays demonstrated a synergistic interaction between the two compounds, with FICI values ranging from 0.236 to 0.5. Notably, vancomycin MICs significantly decreased in combination with 3HL, underscoring their synergistic efficacy against MRSA and addressing a critical gap in AMR research. While all strains exhibited synergy, the FICI values (0.23–0.45) indicated variability in the degree of synergy, suggesting that some strains responded more favorably than others. These findings highlight a novel strategy to enhance the potency of existing antibiotics while exploring complementary mechanisms of action. The combination of vancomycin and 3HL offers multiple therapeutic advantages. The substantial reduction in vancomycin MICs in the presence of 3HL not only signifies synergy but also suggests the potential for reduced dosing, which may minimize adverse effects [12]. Additionally, this combination could help overcome biofilm-related challenges and persistent bacterial infections that are notoriously difficult to treat with monotherapy [34]. 2,3-Dimethylquinoxaline (DMQ) is recognized as a broad-spectrum antimicrobial phytochemical. This study evaluates its toxicological profile through both *in vitro* and *in vivo* methods. Cardiotoxicity, nephrotoxicity, and hepatotoxicity were assessed in cell cultures, while acute oral toxicity (AOT) and subacute oral toxicity (SAOT) were evaluated in mice. Acute dermal toxicity (ADT) tests were conducted in rats. *In vitro* tests showed no significant toxicity at concentrations up to 100 µM, except for a slight, non-significant ATP reduction in human hepatocellular carcinoma cells. The median lethal dose (LD_50_) of DMQ was above 2000 mg/kg, with no mortality or clinical abnormalities observed in animals. Biochemical analysis indicated increased platelet and white blood cell counts by 99.8% and 188.8%, respectively, in treated groups. Histological findings included enlarged renal corpuscles, hyperplasia of testosterone-secreting cells, and coronary and capillary dilation. Overall, DMQ demonstrated an acceptable safety profile in rodents, though high doses caused thrombocytosis, leukocytosis, and tissue alterations, warranting further investigation [35]. Given the structural similarity between 3HL and DMQ, it is reasonable to hypothesize that 3HL may exhibit a comparable safety profile.

While the docking scores and MMGBSA values indicate favorable binding affinities of 3HL and vancomycin with the *mecA* protein, it is essential to recognize the limitations of these computational predictions. In silico methods, though valuable for providing preliminary insights into potential molecular interactions, do not fully account for the dynamic and complex environment within living organisms, such as protein flexibility, cellular uptake, metabolism, and the influence of other biomolecules [36]. Furthermore, high binding affinity in computational models does not always translate to corresponding biological activity *in vitro* or *in vivo* [37, 38]. Therefore, while our docking results support the potential synergistic effect of the drug combination, these findings must be validated through further experimental studies to confirm their biological significance and therapeutic potential.

The combination exploits distinct mechanisms of action: vancomycin inhibits bacterial cell wall synthesis by targeting D-Ala-D-Ala termini, disrupting peptidoglycan crosslinking [39], while 3HL inhibits DNA synthesis and promotes reactive oxygen species (ROS) production [40, 41]. This dual mechanism may explain the enhanced bactericidal activity, as it addresses different aspects of bacterial survival and resistance. The ROS production by 3HL adds an oxidative stress component, further weakening the pathogen’s defenses.

The combination of vancomycin and 3-Hydrazinoquinoxaline-2-thiol (3HL) presents a promising therapeutic strategy in addressing the growing challenge of methicillin-resistant *Staphylococcus aureus* (MRSA) infections. Vancomycin has long been a cornerstone in the treatment of MRSA; however, the emergence of vancomycin-intermediate and vancomycin-resistant *S. aureus* (VISA and VRSA) strains has significantly limited its clinical efficacy [42].

*In silico* docking studies provided further insight into the molecular basis of the observed synergy. Both vancomycin and 3HL effectively targeted key binding sites in PBP2a, a critical enzyme in MRSA’s resistance mechanism. Vancomycin exhibited superior binding affinity (−8.9 kcal/mol) by forming stabilizing hydrogen bonds, while 3HL also demonstrated significant interactions. These findings suggest that the combination targets complementary sites within the protein, potentially enhancing antimicrobial effects through structural inhibition and stabilization. Our docking studies revealed that vancomycin forms stabilizing hydrogen bonds with residues, such as ASN, TYR, THR, and GLU, with donor-acceptor distances ranging from 1.67 to 3.45 Å. Similarly, 3HL (ID: 781248) interacts with residues, including SER, GLN, HIS, LYS, and ASN in various grooves of PBP2a, further reinforcing inhibition of its activity. Notably, vancomycin interacts with critical residues, such as ASN606 and THR373 (within or near the 594–603 region), which are implicated in the enzyme’s function. Hydrogen bond formation, such as the 1.87 Å interaction with ASN606, may help stabilize the active site and prevent loop-mediated occlusion. The complementary binding patterns of vancomycin and 3HL suggest that their combination may disrupt PBP2a’s catalytic function by targeting distinct yet functionally relevant regions, ultimately enhancing antimicrobial efficacy. Our findings suggest that 3HL enhances vancomycin’s antibacterial activity, potentially through synergistic mechanisms that disrupt bacterial cell wall synthesis or target alternative pathways, thereby overcoming resistance. This underscores the need to assess the combination’s activity against VISA and VRSA strains. MRSA infections are often complicated by biofilm formation, which exacerbates antibiotic resistance and hinders treatment. Biofilms serve as protective barriers, limiting antibiotic penetration and shielding bacteria from the host immune response [42]. If the vancomycin-3HL combination proves effective against biofilm-associated MRSA infections, it could represent a significant clinical advancement. Further studies are needed to evaluate its potential in improving bacterial clearance in biofilm-forming MRSA strains. *In vivo* studies will be essential to confirm the efficacy of this combination therapy in biofilm-associated MRSA infections. Understanding its pharmacokinetics and pharmacodynamics will be crucial for optimizing dosing regimens and maximizing therapeutic outcomes. If successful, this novel approach could provide a valuable alternative for clinicians treating multidrug-resistant MRSA infections, particularly when conventional therapies fail. By addressing both vancomycin resistance and biofilm-associated challenges, our study contributes to the development of innovative strategies against antibiotic-resistant pathogens. Future research should focus on evaluating the combination *in vivo* to confirm efficacy and safety, particularly in animal infection models. Time-kill assays will be critical in understanding bacterial eradication kinetics in biofilm-associated infections, while toxicity studies will help ensure the safety of 3HL and its compatibility with vancomycin for clinical application.

## Conclusion

This study demonstrates the synergistic efficacy of vancomycin and 3HL against MRSA, presenting a novel combination therapy that enhances antimicrobial activity while potentially mitigating resistance. The distinct yet complementary mechanisms of action provide a promising strategy for combating β-lactam-resistant bacteria. Future research should explore *in vivo* efficacy, biofilm activity, and toxicity to facilitate clinical translation of these findings.
